# Excavating Early Burawoy: Toward a Third Position in the Race‐Class Debates

**DOI:** 10.1111/1468-4446.70104

**Published:** 2026-03-23

**Authors:** Zachary Levenson, Marcel Paret

**Affiliations:** ^1^ Florida International University and University of Johannesburg Miami Florida USA; ^2^ University of Utah Salt Lake City Utah USA; ^3^ University of Johannesburg Johannesburg South Africa

**Keywords:** Black Marxism, Michael Burawoy, race and class, racial capitalism, W.E.B. Du Bois

## Abstract

This paper intervenes in contemporary sociological debates over the relationship between race and class by excavating the early writings of Michael Burawoy. Against the prevailing polarization between twin absolutist models in which *either* racism *or* capitalism alone possesses causal force, we argue that Burawoy articulates a third position—one that grants relative autonomy to both racism and capitalism while rejecting their causal reduction to one another. Drawing on Burawoy's empirical work in southern Africa from the 1970s and early 1980s, we show how he theorized race and class not as discrete variables, but as articulated through historically specific configurations of labor, state policy, and political struggle. Probing the limits of his formulations—particularly his leanings toward economic determinism and inattention to racial subjectivity—we do what Burawoy himself always advocated: we *reconstruct* his approach. We do this by way of issuing three key injunctions drawn from, but going beyond, his work: (1) interrogate rather than assume the coherence of race and class categories; (2) treat racism as a structured contingency embedded within capitalist social relations; and (3) actively align anti‐racist and anti‐capitalist struggles, moving from logic to strategy. In doing so, we argue that Burawoy offers a distinctively Marxist perspective that does not subordinate race to class, but rather insists upon their mutual articulation. This third position opens the door to a historically situated theory of racial capitalism and a more strategic approach to political struggle.

## 1 A Third Position?

Over the past decade, two significant and related developments have shaped debates among sociologists over the relationship between race and class. One is the belated prominence accorded to W.E.B. Du Bois, thanks in no small part to the efforts of (Morris 2017, 2022), Wright (2016), and others to illuminate Du Bois' foundational contributions after many decades of neglect by the discipline. These efforts include mounting attempts to bring Du Bois—and with him the study of race, colonialism, and white supremacy—into the sanctified space of canonical sociological theory (Itzigsohn and Brown 2020; Burawoy 2021a, 2021b; Meghji 2025). Instructors are increasingly adding Du Bois to their syllabi, alongside Marx, Weber, and Durkheim (Melcher 2024).

A second relevant development is the growing attention to linkages between racism and capitalism, as demonstrated by the increasing prominence of the term “racial capitalism” (Levenson and Paret 2023a). This long ascendent movement to integrate the study of racism and capitalism has arguably been more profound in other disciplines such as American Studies, Black Studies, and geography. But sociologists and sociological journals are becoming increasingly preoccupied with this relationship (e.g., Dantzler et al. 2022; Go 2021; Matlon 2016; Virdee 2019; Wacquant 2024). It is in this context that sociologists of race have discovered Robinson’s (2021) *Black Marxism* nearly 40 years after its initial publication, a book typically cited as a (if not *the*) foundational text on racial capitalism.

These two trends have converged in the form of a debate, bringing together Du Bois and racial capitalism in the form of a question: *was Du Bois a Marxist?* On one side of the debate, Itzigsohn (2023, 2025; see also Itzigsohn and Brown 2020) insists that Du Bois was not a Marxist, but was instead merely influenced by Marxism. In doing so, Itzigsohn draws from Robinson's (2021) argument that Du Bois is best understood not as a Marxist, but rather as a proponent of the Black radical tradition (BRT). For both Itzigsohn and Robinson, to reduce Du Bois to a Marxist would be to undermine the originality of his contribution, which lies in illuminating racism and colonialism as fundamental sources of oppression. As Itzigsohn (2025, 500) puts the point, “if Du Boisianism is really Marxism, then all those challenges [to Eurocentric understandings of modernity] are silenced.” And again: if “Du Bois' thought can be contained within the Marxist tradition,” then there is no possibility of a “distinct Du Boisian sociology” (Itzigsohn 2023, 100). Itzigsohn and Brown (2020) thus call for a Du Boisian sociology that prioritizes the study of “racialized modernity” as constitutive of capitalism and other modern institutions.

On the other side of the debate, Goodwin (2022, 2023a, 2023b) is at pains to demonstrate that Du Bois was, in fact, a Marxist. As evidence, he cites Du Bois' writings from the 1930s onwards, as well as Du Bois' eventual membership in the Communist Party. Whereas Itzigsohn prioritizes racism as the central motivating force of modernity, Goodwin insists that capitalist development should be understood as the primary determinant. In Goodwin's (2022, 2023a, 2023b) reading of Du Bois, racism is not an independent causal force, but is itself a consequence of capitalist development. And against the idea that working within the Marxist tradition undermines Du Bois' originality, Goodwin (2023a, 145–147) suggests that Du Bois and other Black Marxists have been at the forefront of the study of racism and colonialism for over a century.

In many ways, these two positions are mirror images of one another. Each one isolates a key variable, racism or capitalism, as the primary causal force, and then aims to show that the other is best understood as an outcome. But what if a third position were possible, mediating between these twin absolutisms? This is where Burawoy (2022b) enters the debate, presenting an alternative by understanding Du Bois as straddling two distinct dialogs: one with the sociological canon of Marx, Weber, and Durkheim, and another within the tradition of Black Marxism. He refers to this as “walking on two legs” (Burawoy 2022b), rejecting the idea that a scholar may belong to only one theoretical lineage. Given Burawoy's self‐identification as a Marxist, it is not surprising that Itzigsohn (2025) lumps him together with Goodwin. Yet, not only does this conflation distort Burawoy's (2021a, 2022b) own reading of Du Bois, but Itzigsohn's (2025) account largely ignores Burawoy's (1972, 1974, 1976, 1981) early work on the relationship between race and class in colonial and postcolonial contexts. As we will show, this “early Burawoy” avoids *both* the reduction of race to an epiphenomenon of class *and* the treatment of race as fully autonomous from class.

The debates over Du Bois are more than simply an exercise in intellectual history, with the goal of recovering the truth of Du Bois' politics. Rather, they are about identifying the soul of the discipline. In this paper, we put aside the debates over Du Bois and turn instead to the more general questions that undergird them. More specifically, we ask, how should we understand the relationship between race and class, which is to say, racism and capitalism? How are they linked together both in reproducing domination and in articulating organized resistance? We address these questions by excavating the lessons of Burawoy's early work on southern Africa. This includes his analysis of racial and class dynamics in Zambian copper mines immediately following independence (Burawoy 1972a), his comparative study of migrant labor in California and South Africa (Burawoy 1976), and his critique of the “internal colonialism” (Burawoy 1974) and “split labor market” (Burawoy 1981) approaches as articulated in relation to South African history.

Our central argument is that Burawoy represents a third position, distinct from both Itzigsohn and Goodwin, that emphasizes the relative autonomy of race and class. He underscores how capitalist development, articulated through class struggle, propels racism; but he also suggests that racism may operate independently with important consequences for the class structure. If Burawoy hints at a third position, though, his analysis has important limits—not least because it revolves around a political economy of high employment and legalized racism that has largely since disappeared. In what follows, we excavate Burawoy's key theoretical contributions, while also underscore their key limits and what it might entail to extend beyond them. Before turning to Burawoy's early writings on southern Africa, however, we begin by situating them within a broader historical legacy of non‐reductive Marxisms that treat the relationship between race and class dialectically.

## 2 Marxism Without Reduction

Burawoy's early work on race and class did not spring from a vacuum. Rather, it emerged in the wake of both an interwar Black Marxism and a variety of Marxisms that flourished in Africa from the 1960s to the 1980s. It was the latter that had the most profound influence on Burawoy. What united these various tendencies, however, was a non‐reductionist Marxism that treated *both* race *and* class, racism *and* capitalism, as equally important social forces. This non‐reductionist Marxism laid a foundation for Burawoy's theorizing, sometimes directly and sometimes more indirectly.

The well‐known lineage of thinking that many refer to as Black Marxism (including Burawoy 2022a, 2022b, 2024) resonates strongly with the third position that we illuminate by way of early Burawoy. Du Bois was among Black Marxism's earliest proponents, but he was far from alone in developing a dialectical account of the relationship between class formation and racialization. As the Trinidadian Marxist C.L.R. James (1963 [1938], 283) put the point in one of his most widely cited lines, “The race question is subsidiary to the class question in politics, and to think of imperialism in terms of race is disastrous. But to neglect the racial factor as merely incidental is an error only less grave than to make it fundamental.” James was part of a broader Marxist milieu that refused both sorts of determinisms: a racial determinism that failed to historicize race in the context of capitalist development, and a class determinism that reduced race to an epiphenomenon without any causal force of its own.

In this view, “racialized modernity” is inseparable from capitalist development. “Racial antagonism,” insisted Cox (1948, xxx), “is part and parcel of this class struggle, because it developed within the capitalist system as one of its fundamental traits. It may be demonstrated that racial antagonism, as we know it today, never existed in the world before about 1492; moreover, racial feeling developed concomitantly with the development of our modern social system.” Cox too was from Trinidad, as was the historian Eric Williams (2021 [1944], 4), whose landmark *Capitalism and Slavery* begins with a comparable assertion: “Slavery was not born of racism; rather, racism was the consequence of slavery.” From the standpoint of Black Marxism, insofar as we can describe it as a unified position, attributing racial domination to racism was tautological. How could racism account for its own emergence? Black Marxism explained the emergence of racial identification as a consequence of racism, which itself emerged as a component of capitalist development.

Against the notion that the system of racial domination emerged whole cloth from a primordial hostility to difference, Black Marxists are insistent upon historicizing racism itself, which emerged *in relation* to capitalist development. If for Cox racism emerged in relation to imperialism, and for Williams it emerged in relation to the *trans*‐Atlantic slave trade, for Du Bois it emerged much later. As he would put it in *Darkwater*, “The discovery of personal whiteness among the world's peoples is a very modern thing—a nineteenth and twentieth century matter, indeed” (Du Bois 2021 [1920]: 17). And as he would chronicle in much of his subsequent work, and especially *Black Reconstruction*, racism had a class politics, just as class struggle was undeniably racialized.

This was not a matter of causal primacy. Even if racism emerged after (and was indelibly the product of) capitalism, once on the scene, racism was a determinative force in its own right. This was especially true in shaping resistance: “one's social consciousness is determined by one's condition of life, and not the other way around”, as activist Claudia Jones explained (Jones 2024a [1949], 156), riffing on Marx. It was her “Jim Crow experiences as a young Negro woman,” she insisted, that led her to working‐class politics (Jones 2024b [1953], 195). For Jones (2024c [1949], 99), Black women were key to unlocking the potential militancy of Black people more generally, who in turn were key to developing “the anti‐imperialist coalition.” And like James, Cox, and Williams, Jones came from Trinidad. This is no coincidence. In this earlier period, from the 1920s through the 1940s, it was the US and Caribbean—what we might call the Greater Caribbean—that was the key site of the development of Black Marxism.

There is no concrete evidence that Burawoy was reading this early wave of Black Marxism in the 1960s and 1970s. Indeed, he only began to read Du Bois seriously in the early 1990s.^1^ Burawoy was instead much closer to the varied African Marxisms that built upon the foundations laid by Du Bois, James, Cox, Williams, Jones and others. James organized reading circles in London, which kept Marxism and pan‐Africanism in conversation and drew in Jomo Kenyatta, Kwame Nkrumah, and many others who would play key roles in anti‐colonial politics in Africa (Høgsbjerg 2014). Others established important intellectual centers in Southern Africa. From the mid 1960s, the University of Dar es Salaam (UDSM) became just such a center (Al‐Bulushi 2023; Harisch 2025; Zeilig 2022, 166–94), complete with a Caribbean bridge: the Guyanese Black Marxist Walter Rodney taught there on and off from 1966 to 1974. East African Marxists like Ali Mazrui, Issa Shivji, and Mahmood Mamdani influentially theorized class formation in the context of colonial domination, and key Marxist revolutionaries, including Amilcar Cabral and James himself visited the university.

Important theorizing within these circles treated the relationship between precapitalist and capitalist modes of production in African social formations. As Amin (1974, 38) put the point, in the global periphery, “precapitalist modes of production are not destroyed but are transformed and subjected to that mode of production which predominates on a world scale as well as locally—the capitalist mode of production.” Burawoy was particularly taken with Arrighi’s (1970) early account of such articulated modes of production in Rhodesia, and with Wolpe’s (1972) attempt to explain this articulation in South Africa with recourse to racism: racism justified the deliberate underdevelopment of zones maintained as reservoirs of free reproductive labor, which functioned to subsidize the wages of the capitalist labor force.

Indeed, during this same period debates raged in South Africa about the status of race in relation to class formation under colonial rule and then apartheid. In addition to the South African Community Party's (1963 [1962]) introduction of a new program, which thought race and class together as “colonialism of a special type,” key SACP intellectuals published independently on the question. The highly influential *Class and Color in South Africa 1850–1950,* by Simons and Ray Simons (1983 [1969]), suggested, much like Du Bois (1962 [1935]), that racism was a capitalist project designed to fragment the working class. Also influential was Wolpe’s (1972) account of apartheid racism as key to the reproduction of cheap labor‐power. Burawoy was very much alive to the debates about race and class that were taking place within and around the SACP. And just as he was developing his own analysis of racism and capitalism, these debates began to spill beyond Party circles, especially in the critique of racial capitalism developed by heterodox Marxists and pan‐Africanists in the 1970s and 1980s (Levenson and Paret 2023b; Paret and Levenson 2025).

These intellectual connections reflected actual relationships. In the wake of the Sharpeville Massacre, the Simonses received banning orders from the apartheid state, and in 1967, they went into exile in Zambia. Both had been instrumental in reviving the SACP after a period of dormancy in the 1930s. Jack Simons would go on to teach social science at the University of Zambia, where he launched an MA program in sociology along with fellow Marxists Jaap van Velsen and Raja Jayaraman. Burawoy was the first student to complete their grueling Marxist bootcamp, producing along the way an MA thesis on student protest (Burawoy 1972b, 1976) and a study of race and class on the Zambian copper mines (Burawoy 1972a). And so, while discussions about “colonialism of a special type” played out in South Africa, and debates over “African socialism” raged in Tanzania, Burawoy, Simons, Bernard Magubane, and quite a few others were in Lusaka trying to make sense of the relationship between systems of racist domination and class formation in the context of decolonization. Burawoy later became close friends with Wolpe after Burawoy's dissertation advisor at the University of Chicago, William Julius Wilson,^2^ invited Wolpe to visit in the 1970s. As Webster et al. (2024) recount, “regular visits and extended conversations with Wolpe had a major influence on Burawoy's understanding of South Africa.” Situating race, caste, and migration in relation to capitalism, van Velsen, Jayaraman, and Wolpe helped to inspire Burawoy's early work (see also Burawoy 2004).

While Burawoy was clearly working broadly in this non‐reductive tradition, there were quite a few missed connections we find surprising. For example, he remained isolated from the debates in the Dar School at the time, despite the key influence of Arrighi, who taught at UDSM in the late 1960s. One of the most significant missed encounters is the case of Stuart Hall and the broader Black British Cultural Studies tradition, which Burawoy did not encounter until well after his work on southern Africa (see Burawoy 2022b, 583). It is especially curious because Burawoy frequently traveled to the UK, his birthplace, and because Burawoy and Hall both pointed to the “relative autonomy” of race and class. Their main point of connection appears to be Wolpe, who profoundly influenced both Burawoy's (1976) understanding of migration and Hall's (1980) conception of articulation.

Similar to Hall, Burawoy would find theoretical foundations in Gramsci's (1971) *Prison Notebooks*, which he discovered in the mid‐1970s under the tutelage of Adam Przeworski. This produced some remarkable convergences between these two thinkers. One of Hall et al.'s. (2013 [1978], 386) and Hall's (2021b [1978], 65; 2019 [1980], 216; 2016 [1983], 149) most widely cited formulations, that race is the “modality” or “prism” through which class is lived,” first appears in print six years after Burawoy (1972a, 115; see also his similar use of “idiom” in 1972b, v, 8, 23) described class struggles as “cast in the idiom of race,” a formulation discussed below. Likewise, Hall (2021a [1977]: 121) is well known for his formulation that “[t]he superstructural results can ‘react’ upon the base.” Three years earlier, Burawoy (1974, 542) would describe the effectivity of the superstructure in nearly identical terms, “in turn react[ing] back and modify[ing] the economic base.” The resonance is striking, though it appears to be purely coincidental. Still, we cannot help but wonder if nearly identical language was a product of close readings of similar debates, including Gramsci and Wolpe, as well as Poulantzas and Althusser.

Insofar as there was a key figure influencing Burawoy in this period, it was undoubtedly Frantz Fanon (e.g., Burawoy 2014, 968). In particular, Burawoy was interested in both embracing and exploding monolithic categories like “the colonized,” which Fanon (1962) achieved in *Wretched of the Earth* when he showed how the formation of a national bourgeoisie fragments racialized groups, enabling cross‐class coalitions. This is of course deeply resonant with *Black Reconstruction*, in which Du Bois (1962 [1935]) pioneered the study of racialized class fractions and the formation of multiclass alliances. Whereas Du Bois (1962 [1935]) laments the racial divide within the working class, Fanon (1962) bemoans the way in which the national bourgeoisie captures the process of decolonization. Both underscore the complex articulation of racial and class forces, and ultimately, how they undermine progressive social transformation. Such complexity constitutes Burawoy's point of departure: treatment of the empirical world in all of its messy reality rather than reducing it to theoreticist abstractions.

## 3 Class and Race Are Not Automatic Unities

At the core of Burawoy's approach are complex understandings of race and class, and thus a refusal to treat either category as necessarily uniform or inherently unified. He develops such an understanding through critiques of internal colonialism (Burawoy 1974) and split labor market (Burawoy 1981) approaches, which were prevalent at the time. Burawoy (1974, 524) argued that the former “virtually ignores divisions within the black community.” In South Africa, this was evident in the South African Communist Party's (1962) manifesto on “colonialism of a special type,” which argued that, “There are no acute or antagonistic class divisions at present among the African people.” Likewise, Bonacich’s (1972, 1976, 1981) model of the split labor market assumed both the uniform interests of capital, downplaying sectoral divisions (e.g., manufacturing vs. mining capital), as well as the uniform interests of white workers, who were nonetheless divided by skill level.

In practice, however, class differentiation did exist within each ethno‐racial category. As Burawoy (1981, 291) makes the point in the context of Northern Rhodesia (Zambia),

long before political independence, class conflict had developed within the African population, between the mass of unskilled workers, who were mainly concerned with reducing the level of exploitation, and the skilled, supervisory, and salaried staff Africans, who were more interested in eliminating color bars that reserved jobs for whites.

As he explains, each race‐class fraction had notably distinct political interests: Black unskilled workers fought to reduce the rate of exploitation, whereas Black managerial workers directed their energies toward contesting the reservation of jobs for white workers—their direct competitors, unlike the proletarian masses. Turning to colonial and apartheid South Africa, which Burawoy (1981) reads through a critique of Bonacich's split labor market theory, he examines crucial divisions among white workers. Whereas skilled white workers sought to maintain the color bar and thus their monopoly on skills, unskilled white workers, who were threatened by replacement by Black workers, preferred to eliminate the color bar and thus exclude Blacks from employment entirely (Burawoy 1981, 302; 2021a, 2021b, 115–118). Mirroring this divide were sectoral divisions between mining, agricultural, and manufacturing capital, all entirely white but with divergent economic interests. Mining and agriculture sought to secure cheap Black labor through color bars and the racially oppressive migrant labor system, and thus competed for migrant workers. Conversely, the interests of manufacturing capital lay in the stabilization, and thus upward mobility, of Black workers (Burawoy 1981, 294–295, 317–318).

At the root of Burawoy's approach is a rejection of *both* simplistic race *and* simplistic class narratives, which treat either racial or class groups as uniform actors with common interests. Class divides racial groups, and race divides classes. Both are further fragmented by skill level, industrial sector, and ethnonational identity, among other factors. This means that neither broad classes such as workers or capitalists, nor racial groups such as Black and white, have uniform economic interests that we can know in advance. Nor can we say, for example, that particular race‐class fractions, such as white workers, are necessarily unified. Rather, class, race, skill, and sector articulate in specific configurations in each place and historical moment, shaping the interests of particular groups and the alliances that follow. In Burawoy's analyses of Zambia (Burawoy 1972a) and South Africa (Burawoy 1974, 1981), we thus see a proliferation of groups defined by *combined* characteristics: dispossessed Black peasants; unskilled Black workers; skilled white workers; white supervisors; poor whites; Afrikaner farmers; British‐owned mining companies; manufacturing capital; white landowners; the Black bourgeoisie and middle class; and so on.

By demonstrating these divergent interests empirically, Burawoy (1972a, 114) is revealing the limits of “a simple two‐class model,” replacing it with one that takes seriously “the interaction of race and class as conceptually distinct categorizations.” An overly simplistic Marxism then assumes a movement toward class unity, which is to say that working‐class people unite across racial lines. But as he shows in the context of pre‐apartheid South Africa, “inclinations toward inter‐racial solidarity gave way to inter‐class alliances and the exacerbation of racial divisions” (Burawoy 1974, 535). Much as in Du Bois' *Black Reconstruction*, white workers forged alliances with white managers and even white capitalists, rather than their fellow workers.

Rather than “a division *between classes*,” then, we encounter “a division *within a class*” (1974, 542). As Burawoy argues, we cannot map uniform interests onto monolithic racial groups. Rather, the material interests of each racial group vary in accordance with what he describes as their relation to the “colonial superstructure.” “Economic interests,” he argues, “are determined not only by class interest, but also by the existing institutional framework and ideology, that is by relations to the superstructure as well as to the means of production” (Burawoy 1974, 528). This leads Burawoy (1981) to a Du Boisian understanding of race. If Du Bois (2014 [1940]) famously argued that “the black man is a person who must ride ‘Jim Crow’ in Georgia,” Burawoy (1981, 280) argues that “no longer does one have few resources because one is black; instead one is black because one has few resources.” Here he is challenging Bonacich's suggestion that race determines one's access to resources, suggesting instead that we view race as a dependent variable, an effect of state policy and the division of labor.

Theorizing race as “superstructural” in this context—Burawoy's preferred terminology in this early work—by no means suggests a lack of causal effectivity. In fact, Burawoy (1974, 542) is explicit: “new institutional mechanisms in the superstructure…in turn react back and modify the economic base.” In other words, racism was articulated through political institutions, which fragmented each class into segments, each of which was inserted differently into the relations of production. And so, racism quite clearly shapes the relations of production and, therefore, the specific articulation assumed by capitalism in any given conjuncture. In the case of South Africa, a racist superstructure bound together Afrikaners across class lines rather than the working class across racial lines: “Economic interests of black and white segments of the lower classes diverged because of their differing relations to the superstructure. The prevailing ideology of white supremacy and the institutional framework which discriminated on the basis of color, militated against inter‐racial solidarity and led to shared interests between landed and landless Afrikaners” (Burawoy 1974, 537).

## 4 Capitalism and Racism: A Dialectical Feedback Loop

If Burawoy's first step was to expose the internal diversity of both racial and class categories, his next step was to consider the dynamic relation between racism and capitalism. For Burawoy, the relation runs in both directions: class struggle drives racial conflict, and racial domination reproduces capital accumulation. Capitalism and racism are therefore inextricably linked, mutually imbricated in a dialectical feedback loop.

Burawoy's analysis of the Zambian copper mines reveals the class dynamics that lay beneath the reproduction of the “color bar”—the colonial era policy of ensuring that white workers always had higher status than Black workers within the workplace hierarchy. Why did the color bar persist after Zambia achieved self‐rule, and despite efforts to increase the presence of Black workers in jobs previously reserved for white workers? Burawoy (1972a, 2014) unearths the class content of a phenomenon that appears to be purely racial. Most Black workers were concerned with better wages and working conditions, rather than upward mobility. White managers sought to maintain their privileged positions. Corporate management wanted to preserve stability by keeping these managers happy. And representatives of the postcolonial state—presumably a key force in favor of “Zambianization,” or the replacement of expatriate workers with Zambians—were more concerned with maintaining export revenue. They also worried that upwardly mobile Zambians might constitute an effective political opposition (Burawoy 1972a). As Burawoy (2021b, 73) would later reflect, “In moving from micro‐processes to their macro‐foundations, I was able to identify the class interests behind the postcolonial racial order.” Attention to the capitalist foundations of persistent racism thus reveals a “paradox—class interests were responsible for the postcolonial reproduction of a colonial racial order” (Burawoy 2014, 967).

Burawoy (1974, 1981) follows a similar script in his analysis of South Africa, which examines the unfolding of colonial and apartheid rule over the first three quarters of the twentieth century. At the center of his analysis are the ways that class struggles shape racist state policy, from the “civilized labor policy” of 1924, designed to bolster the employment of poor whites, to the rise of apartheid in 1948, which would cement the super‐exploitation of Black workers. In each case, it is a series of class alliances that enables state racism. The “civilized labor policy” was a response to the agitations of white workers and landed Afrikaners, and also benefitted all fractions of capital by undermining an emergent inter‐racial working‐class solidarity (Burawoy 1981, 307–311). Apartheid reflected a white alliance across class lines, from mining and agricultural capital to lower‐class Afrikaners, including the petty bourgeoise and working class. This emerges against the backdrop of burgeoning Black militancy, facilitating a counter‐alliance between the Black working class and petty bourgeoisie. The Afrikaner nationalist alliance won out over the interests of manufacturing capital, which sought a more liberal racial order (Burawoy 1981, 315–319).

What, then, is the role of racism and racial ideology? In his first book, Burawoy (1972a, 115) argues that class struggles were “cast in the idiom of race.” Race functioned in this case “as the idiom of conflict and co‐operation” (Burawoy 1972a, 115). Subsequently he would use the imagery of “terrain” to describe this phenomenon:

Where institutionalized racism invades every corner of social life, class struggles can only be fought out on the terrain of racism. However, precisely how that struggle will be fought and with what consequences depends upon the balance of class forces within each racial group and the form of the apartheid state.

(Burawoy 1981, 324)

But in either case, as idiom or terrain, race works to *express* class. But at the same time, Burawoy argued that race *obscures* class. Racism does “not, per se, alter the class structure of society; rather [it] tends to obscure it” (Burawoy 1972a, 117). Likewise, a decade later he was still maintaining that racism “conceals” the class structure:

It is precisely because “race relations” and “racial antagonisms” do conceal such diverse interests that “racism” is such a powerful ideology, binding together classes within racial groups, shaping class interests, promoting particular configurations of alliances, and generally shaping the terrain and expression of class struggle.

(Burawoy 1981, 292)

And so for Burawoy, race and class are locked into a contradictory relationship. Even as race may “conceal” or “obscure” productive relations, it also articulates these very relations: “shaping the terrain and expression of class struggle.”

In all of these examples, if class struggle reproduces racism in the form of color bars and apartheid, then the latter shore up capital accumulation by reproducing exploitation, and especially the super‐exploitation of Black workers. Capitalism and racism thus develop in tandem. Burawoy's (2021b, 92) conclusion, as he would elaborate in retrospect, was that “you can't study race without attending to class, and that you can't study the political superstructures that constituted race without attending to the dynamics of capitalism … capitalism, far from being impacted [undermined] by a racialized superstructure, could effectively expand within and through institutional racism.”

Underpinning this analysis is a sophisticated understanding of “interests.” As Burawoy (1974, 528) remarks, economic interests are “determined not only by class interest but also by the existing institutional framework and ideology, that is by relations to the superstructure as well as to the means of production.” This is an approach to Marxism quite distinct from the version that reduces material interest to an automatic outcome of location in the class structure. Here, Burawoy (1974, 542) is carefully distinguishing economic interests from class interests, identifying ways that economic interests are mediated by “new institutional mechanisms in the superstructure which in turn react back and modify the economic base.” In laying out this approach, Burawoy (1974) thus distinguishes between three distinct objects of analysis: racial interests, as defined by the state; class interests, as defined by the relations of production; and economic interests, which represent a combination of the two. This affords a relative autonomy, and thus causal force, to both class and race, which both have real, material effects that can be “very difficult to disentangle” (Burawoy 1972a, 23). For Burawoy, we do not need to choose between either racism or capitalism as the primary driver of economic interests. Rather, economic interests emerge from a *combination* of both forces.

## 5 The State Guarantees the Reproduction of Racial Capitalism

The state lies at the center of Burawoy's analysis of southern Africa, and of his theoretical understanding of the relationship between race and class more broadly. This is because, in his view, the state does two things at once: it is the primary agent of capitalist reproduction, securing the interests of capital; and it generates racial categories and exclusions. It follows that Burawoy's (1981) central critique of Bonacich is her elision of the state, which explains her inability to reconcile both the political interests of capital as a class, and the systemic character of capitalism as a mode of production (Burawoy 1981, 284). This critique of Bonacich illuminates how the state facilitates the articulation of capitalism and racism, what he would subsequently describe as “racial capitalism” (Burawoy 2021b, 113–119).

Following Johnstone (1976), Burawoy (1981, 304) illuminates the ways in which the South African state organized the class/race linkage by pointing to two different sets of mechanisms: “exploitation color bars, which are a form of domination of capital over black labor, and job color bars, which secure the domination of white labor power over black labor power.” The “exploitation color bars” refer to the apartheid pass laws, which regulated the geographic mobility and employment of Black workers, the restriction of their political rights, and their housing within oppressive compound structures defined by surveillance (see also Burawoy 1976; Paret 2024). These mechanisms ensured “the particular powerlessness and cheapness” of Black workers (Burawoy 1981, 304). Conversely, the “job color bars” included laws that reserved skilled and supervisory jobs for white workers, as well as the preferential access of unskilled white workers to public employment, the so‐called “civilized labor policy” (Burawoy 1981, 304). Taken together, these various “color bars” ensured despotic control in the workplace, giving white workers carte blanche to dominate Black workers (von Holdt 2003).

In his critiques of both Bonacich and theorists of internal colonialism, Burawoy (1974, 1981) trains his attention on the state. In one of his better‐known essays on migrant labor (Burawoy 1976), however, he emphasizes how these mechanisms ensure the spatial and economic organization of race/class articulation. According to Burawoy (1976), a migrant labor system revolves around the geographic separation of labor force *maintenance*, or the daily reproduction of the worker, and labor force *renewal* via the reproductive activities of the household and community. Within this system, racial identity and economic organization become indistinguishable. This is precisely what Arrighi, Wolpe, and others were getting at: race serves as both a mechanism and a justification for the development of underdevelopment, which is to say the reproduction of so‐called “natural” economies left unscathed by proletarianization, which provide subsistence to families at no cost to employers. The spatial arrangement is key, hence *migrant* labor. Formally employed workers leave these “natural” economies, as Luxemburg (2015 [1913]) called them, to work in properly capitalist zones, and then return to their villages to recuperate—Burawoy's renewal. Production takes place under capitalism, while renewal occurs in a non‐capitalist zone. These two zones map onto two distinct geographic spaces, which serves to lower the cost of reproducing wage laborers and their families.

If the geographic separation of production and reproduction, which facilitates the lowering of wages, defines the migrant labor system, it is nonetheless the state that ensures the reproduction of the system as a whole. Burawoy (1974, 1981) borrows this point from Wolpe (1972), who argued that apartheid represented an attempt by the state to salvage a cheap labor system that was unraveling as proletarianization undermined “non‐capitalist” zones of renewal in rural areas. The centrality of the state thus highlights the complexity of the “cheapness” of labor. Burawoy (1976, 1055–1057) poses the question succinctly: cheap for whom, employers or the state? Not only does the state incur significant economic costs in reproducing a migrant labor system, via exploitation color bars and job color bars, but it must also manage the political consequences of its actions. From the 1970s through its 1994 demise, the apartheid regime confronted significant resistance both domestically and internationally, which only reinforced economic strains. These events hint at the need to develop a dynamic theory of the state.

## 6 A Conjunctural Analysis of Racial Capitalism

Given Burawoy's emphasis on concrete articulations of race and class, it is not surprising that periodization and conjunctural analysis were central to his analysis. Rather than a generalized, transhistorical racism, he argues that “racism is understood as the product of particular developments in the capitalist economy” (Burawoy 1981, 281) In this respect, Burawoy's well‐known critique of Edna Bonacich has much in common with Stuart Hall's roughly contemporaneous critique of Harold Wolpe. If Wolpe (1975, 117) argued that South African capitalism “necessarily require[s]…colonial racism” (see also Wolpe 1972), Hall (2019 [1980]) countered that this was only one possible solution, and that Wolpe's account was essentially functionalist, premised upon a logical solution to a historical problem. Instead, Hall (2019 [1980]: 209) insisted, we need to “grasp the structurally different relations in which ‘white’ and ‘black’ labor stand in relation to capital.” Put simply, proclaiming that racism fragments the working class does not get us very far. As Burawoy (1981, 281) points out, the logical fact that labor market dualism is functional for accumulation does not tell us how it develops differently, if at all, in different geographic contexts. Rather than assuming a necessary relation, as did Wolpe (1972, 1975), we must instead explain the existence of racism and trace its genesis (Burawoy 1981, 315). And to do this, Burawoy (1981, 293) suggests, we need to understand processes of racial (and class) formation within “the specific class structure and state in which they are embedded.”

Burawoy's (1981) critique of Bonacich is rooted in a plea for greater attention to the political bases of class interests. He takes Bonacich to task for understanding capital‐in‐general as if it were an individual capitalist writ large, pursuing its immediate, profit‐driven interests. This ignores the fact that different segments of the capitalist class both collaborate and compete and, in the process, produce novel articulations of racial and class domination. Put differently, Bonacich writes the politics of group formation out of her narrative, which for Burawoy is a politics that is ultimately worked out within and through the state: “The emergence of the unique social formation which laid the basis for subsequent economic and political development of South Africa can only be understood as the outcome of class struggles objectified in and organized by the state” (Burawoy 1981, 300).

It is the politics of group formation and struggle in each particular conjuncture, therefore, that transforms systems of domination. This is why Burawoy (1974, 1981) is adamant that one must not reduce South African history to a uniform racial capitalism that persists over time. It is the leading alliance of forces, the specific racist policies they promoted, and the connection of those policies to the relations of production, that defines each period. In revisiting this analysis, Burawoy summarizes the dynamic in the following way:

Here one has to examine the *capacity* of different groups to enforce their interests, both separately and through alliances—interests that come to be expressed in state intervention even as those interests are themselves constituted by the state. A particular fraction of the dominant class becomes hegemonic, forging a temporary unity both *within* the dominant class as well as *over* the dominated classes. Impelled by the dynamics of capitalism, however, each hegemonic system enters into crisis to be replaced, sooner or later, by another hegemonic order reflecting a different coalition of classes.

(Burawoy 2021a, 2021b, 117, emphasis in original)

In South Africa, the leading alliance tended to alternate between representatives of capital and a white nationalist alliance. Each one had implications for the contours of state racism and the division of labor (Burawoy 1981, 319–320).

This analysis appears to focus on class, but what about race? Burawoy (1974, 529) is clear that the relationship between racist state policy and the capitalist economy is “one of both conflict and cooperation. Stressing conflict tends to explain change but not persistence, while stressing cooperation tends to explain persistence but not change.” The key, then, is to understand how racism either advances or undermines the interests of certain classes or class fractions.

But this framing of conflict and cooperation is too rigid. It suggests that class rests in the economy while race lies within the state, whereas the reality is far more complex. Burawoy’s (1981) subsequent analysis of South Africa moves beyond this cruder formulation, published 7 years prior (Burawoy 1974). In his later analysis, Burawoy’s (1981) is clear that racial and class struggles are deeply intertwined. On the one hand, the struggle against white supremacy can only be effective if it is anti‐capitalist: “The struggle for national liberation, for the end of racism, or even the mitigation of racism is a struggle against capitalism” (Burawoy 1981, 324). This is because state racism held together both the labor process and the reproduction of labor power, thus ensuring that “the survival of South African capitalism rests on the survival of South African racism” (Burawoy 1981, 324). On the other hand, it follows that a racialist conceptual apparatus and racist laws play a central role in class struggles. As Burawoy (1981, 324) puts the point, “Where institutionalized racism invades every corner of social life, class struggles can only be fought out on the terrain of racism.”

## 7 Reconstructing Burawoy: Toward a Research Agenda on Race and Class

Despite immense contributions to our understanding of the linkages between race/racism and class/capitalism, Burawoy's analysis is not without its limits. First and foremost, he was writing about a context in the 1960s and 1970s that differs significantly from the present. For example, his analysis pertains to a context where employment opportunities were relatively abundant. Today, the expanded unemployment rate, including “discouraged” job‐seekers who have given up searching for work, hovers around 40% in South Africa. Likewise, Burawoy’s (1974, 1974, 1981) analysis of South Africa focuses on a situation of legalized racism that was largely delegitimated by the close of the 20^th^ century—with the obvious exception of Israel (Burawoy 2025). If racial inequality and racial domination persist, they do so via a political apparatus that appears very different from the one Burawoy examined. In the period from the late twentieth to the first quarter of the twenty‐first centuries, states tended to distance themselves from explicit racism, rather than legalizing and endorsing it (Winant 1994, 128).

Beyond these contextual limits, we may also point to the limits of Burawoy's theoretical approach. This includes a whiff of economic determinism. At times in Burawoy's analysis, racial domination appears as a mere intermediary that secures the tight connection between capitalist interests and profit‐oriented state policy. He was grasping for an approach that afforded relative autonomy and causal force to both race and class, with the former rooted in the state or “superstructure,” and the latter within the division of labor or the “economic base”:

[T]he superstructure emerges out of, interacts with and modifies the economic base … Though the superstructure is a reflection of the economic base, the former is in no way uniquely determined by the latter … while the superstructure corresponds to and its development is constrained by the economic base, it is at the same time the unique product of historical events and economic change.

(Burawoy 1974, 545)

But we can also see Burawoy wavering here, between emphasizing the contingency of the relationship between racism and capitalism, and a cruder view that presents the racist state as “reflecting” or “corresponding to” the “economic base.” It is no wonder that, in reflecting on his southern Africa writings, he suggests that the chink in the armor of 1970s Marxism was its propensity for functionalism (Burawoy 2021b, 119). An adequate approach must dispense with all functionalist formulations.

We must also extend Burawoy's understanding of race. The state, which lies at the center of his analysis, remains a crucial race‐making institution, though one that is most effective when official categories align with popular understandings (Loveman 2014, 11–23). In addition to the state, a thorough account of the linkages between racism and capitalism must attend to cultural and emotional investments in racial identification. This is Burawoy's Achilles' heel. He provides an impressively detailed analysis of the labor process, the mode of production, the class structure, and racist state institutions.^3^ But he pays less attention to the subjective dimensions of racialization. This limit is especially significant in the contemporary period, where the retreat of the state from formal, explicit, legalized racism amplifies the significance of society and culture within racialization processes. We can push beyond Burawoy's analysis by paying greater attention to lived experiences of race, including processes and experiences of dehumanization that often catalyze popular struggles.

With these limits in mind, we present three injunctions for making sense of the relationship between race and class. Each one draws inspiration from Burawoy's analysis as characterized above. Our goal is to distill the insights of his analysis, but also to reconstruct and extend his approach, addressing the aforementioned limits, to make it relevant to our present moment.

Injunction 1 Interrogate categories. Do not take race and class for granted as collectives with uniform interests. Rather, unpack the interests of particular groups within concrete institutional and ideological configurations.

Burawoy usefully illustrates that neither race nor class is a monolith. Both are internally diverse and potentially causal. Methodologically, this pushes us to root our analysis in concrete historical situations defined by specific capitalist relations, state policies, and ideologies. Burawoy focuses heavily on the labor process, which accounts for the prominence of skill‐based divisions in his analysis. This is a promising line of inquiry. The benefits are evident, as Burawoy is able to demonstrate the different interests at play within southern African capitalism, and how those interests propel changes within the workplace and the state. But we must also look further, beyond the labor process, to varying institutions such as the housing market, border enforcement, and the church. These institutions are fluid, and therefore so will be the interests of particular classes and racial groups. The key take‐away is that analysts must take care not to assume the uniformity of racial or class interests. Instead, we must train our analytic lens on the concrete level and explicitly specify the sources or mechanisms of domination and oppression.

Injunction 2 Racism is a structured contingency. Situate racism within capitalist social relations.

Burawoy places capitalism at the center of his analysis of racism. Indeed, he takes theorists of racism to task for failing to recognize that race and racism are “forged in the fire of capitalism” (Burawoy 1974, 90). In challenging Bonacich's decision to take racial division as a point of departure, he argues that “a more realistic approach to power involves taking class relations as a *point of departure* for the study of race relations … this does *not* mean that we ignore either [race or class], but it does mean that we study racial antagonism within the context of capitalist relations of production” (, 315, emphasis in original). Through his analysis of South Africa, we can see how racism may become functional for capitalism, such as by fragmenting the working‐class and enabling the hyper‐exploitation of Black workers.

But racism and capitalism are never *necessarily* reinforcing. As Burawoy (1981, 529) himself underscores, they may cooperate or come into conflict. This is not a necessary relationship but a *contingent* one. Dispensing with functionalist arguments, we prefer instead to examine how the articulation of different institutions sets limits on, and encourages certain patterns of, group formation and struggle. This is what Stuart Hall (2021c [1983], 153) refers to as “tendential lines of force.” Ultimately, avoiding functionalism and a crude determinism is about embracing what we might call a *structured* contingency—contingency in the sense that outcomes are never fully determined, but structured in the sense that capitalist class relations set limits on possibilities for racial formation. This is not about capitalism “trumping” racism in its explanatory power; it is about acknowledging that racism is not some primordial force that exceeds history (Burawoy 2021b).

Injunction 3 Align anti‐racist and anti‐capitalist struggles across multiple terrains.

Burawoy offers two key insights with respect to strategies of resistance: first, in racially‐divided societies, class struggles necessarily take place on the terrain of race; and second, effective anti‐racism requires a challenge to capitalism. The implication is that anti‐racist and anti‐capitalist struggles are not opposed, and indeed, they tend to require each other. But this is far from a foregone conclusion. In South Africa, the African National Congress and its alliance partner, the South African Communist Party, promoted a two‐stage revolution that explicitly decoupled anti‐racism and anti‐capitalism. Such developments remind us that we should pay careful attention to the “class composition of the nationalist [or antiracist] alliance,” a point that Burawoy (1981, 325–326) recognized, even if his optimism about the possibilities for a “prototypical Marxian revolution” blinded him to the conservatism of an emergent nationalist bourgeoisie. A key take‐away is that effective resistance will likely require active efforts to align anti‐racist and anti‐capitalist mobilization. These two modes of anti‐systemic struggle *can* be articulated into a singular program, but this is never automatic. It takes a particular theoretical orientation underpinning one's approach to organizing.

It is crucial, however, that we do not reduce anti‐racist struggles to anti‐capitalist struggles. Racialization is deeply felt, and without attention to the lived experience of race, we risk reversion to a functionalist mode of argumentation in which anti‐racism solves a *logical* problem for anti‐capitalist struggle—how to unite the working class—without simultaneously posing this question as a *strategic* one: how to secure emotional investment in process. Burawoy was serious when he suggested that anti‐capitalist struggles are frequently articulated in the idiom of race. This means that the question of class formation is inextricable from processes of racial formation. And to be clear, this formulation challenges both absolutisms with which we opened this paper. On the one hand, it calls into question any anti‐racist strategy divorced from anti‐capitalism, an approach that assumes the unity of racial groups in advance. On the other, it poses a problem for a reflectionist Marxism that wants to read racial identification directly from the economic base. It was Marx (1970 [1859], 21), after all, who insisted that it is in the “ideological forms in which men become conscious of this conflict and fight it out.” And so understanding class struggle without working through the relative autonomy of these “ideological forms” likewise proceeds from logical assumptions, rather than historical and strategic analysis. Our alternative is to work through the concrete ways in which anti‐racist and anti‐capitalist struggle are linked.

## 8 Conclusion

As we have demonstrated here, Burawoy's position is distinct from both Goodwin's and Itzigsohn's. If Itzigsohn consistently argues for a non‐Marxist Du Bois, he is doing so in the name of highlighting the explanatory power of racism. In calling for a Du Boisian sociology, he suggests that any affiliation with Marxism will demote the causal priority of race and racism. On the other side of the aisle, Goodwin is exemplary of a Marxist impulse to incorporate Du Bois, ultimately in the name of subsuming the driving force of racism under the weight of capitalist determination. Burawoy, meanwhile, refuses to select one—racism *or* capitalism, race *or* class—as the most important “variable,” pointing instead to their varied articulations within particular conjunctures. While we appreciate parsimonious explanations, in this particular case we seem to be at an impasse. Yes, Goodwin is correct that Du Bois' analysis became increasingly Marxist over time, and he's equally correct that Du Bois roots modern racism in the process of capitalist development. But the way he advances his argument downgrades racism to a subsidiary category, essentially rendering it an *effect* of capitalism. This strips racism of its relative autonomy, which is to say, that once racism arrives on the scene, it may or may not follow capitalist imperatives—a point that Burawoy makes very clearly.

Burawoy is equally resolute: racism is not some primordial impulse, but rather emerges in concrete socio‐historical contexts. And as Du Bois argued quite clearly, these contexts are invariably capitalist. This is the sense in which we agree that Du Bois is absolutely a Marxist, but of a very particular sort. In a late lecture, Burawoy (2024) developed an understanding of Black Marxism that avoids two pitfalls: first, it avoids subsuming racism under capitalism in a narrow causal sense; and second, it refrains from effacing capitalism from the story of racism's reproduction. Instead, he argued, the tradition of actually existing Black Marxism—and he named a few, including Du Bois, C. L. R. James, Frantz Fanon, and Stuart Hall—charts a third path. It remains Marxist insofar as it retains Du Bois' emphasis on the capitalist underpinnings of racism. But “Black” here signals the relative autonomy of race. To be clear, “relative autonomy” is not intended as an escape hatch for Marxists seeking to rebut charges that they downplay the determinative role of racism. Rather, as Burawoy's early work shows us clearly, the accent is on *autonomy*: once in place, racism (or anti‐racism) can reshape capitalism with just as much impact as capitalism can propel racism. In our view, some of the most promising contemporary scholarship on race and class maintains this emphasis on relative autonomy by situating racism within capitalism without diminishing the determinative power of race (e.g., Bhattacharyya 2018; Clarno 2018; Go 2024)—as does our own work (e.g., Levenson and Paret 2023b; Paret and Levenson 2024).

Burawoy's third position is a crucial corrective, allowing us to make sense of society as it actually exists rather than attempting to force it into our preconceived theoretical predilections. Whether ethnographic or historical, he begins with a theoretical framework—Marxism—but then allows the empirical world to challenge, stretch, and ultimately, to *reconstruct* that framework. This is, perhaps, Burawoy's key contribution: his insistence on rooting the study of race and class in lived experiences and concrete historical formations. Doing so allows us to remain open to different configurations and pathways of causality. The alternative is a hardline deductivism—what Burawoy called “theoreticism”—that runs roughshod over the empirical world, smothering it until the only sound left is a single syllable: race or class. Following Burawoy, we prefer to understand race and class as equally important and enduringly linked, but in ways that are constantly shifting and subject to contestation.

## Funding

The authors have nothing to report.

## Ethics Statement

The authors have nothing to report.

## Consent

The authors have nothing to report.

## Conflicts of Interest

The authors declare no conflicts of interest.

## Permission to Reproduce Material From Other Sources

The authors have nothing to report.

## Data Availability

Data sharing not applicable to this article as no datasets were generated or analysed during the current study.

## Endnotes

^1^
Burawoy would not read *Black Reconstruction* for many decades, nor was Du Bois a formative influence in his earliest period of writing. In fact, he would not read Du Bois at all until the early 1990s (personal communication, July 19, 2024). He did not even encounter Du Bois in graduate school while studying race under the supervision of William Julius Wilson (Meghji et al. 2024, 6). It is very likely that Burawoy did not turn to *Black Reconstruction* until the 2010s. On his comparison of Du Bois and Fanon, see Burawoy (2022a, 2024).

^2^
It was in precisely these years that Wilson (2012 [1978]) was working on *The Declining Significance of Race*, a fact we had not considered until working on this paper. Over 40 years later, Burawoy (2021b, 53) summarized that book as follows: “The civil rights movement, dominated by middle‐class Blacks fighting for inclusion through such policies as affirmative action, largely overlooked the impoverishment of an increasingly marginalized and destitute population of African Americans.” While Wilson's project is quite different from Burawoy's, we cannot help but note the Fanonian resonance here: both sociologists wanted to understand how class formation fragmented racial groups.

^3^
Burawoy's work emerged in an intellectual climate informed by structuralist analyses of racism. In other words, even work that focused on contextual specificity in this period tended not to consider emotional investment in racial identity as a key factor. This is not to excuse the absence, but rather to contextualize his writing. An argument might be made that emotional investment was more central to the first wave of Black Marxists in the first half of the twentieth century, but that the second wave, largely rooted in Africa and the UK, were deeply by influenced structuralist Marxism and debates adjacent to dependency theory—as was Burawoy.
